# Molecular Self-Organization in Surfactant Atmospheric Aerosol Proxies

**DOI:** 10.1021/acs.accounts.3c00194

**Published:** 2023-09-09

**Authors:** Adam Milsom, Adam M. Squires, Andrew D. Ward, Christian Pfrang

**Affiliations:** † School of Geography, Earth and Environmental Sciences, University of Birmingham, Edgbaston, Birmingham B15 2TT, U.K.; ‡ Department of Chemistry, University of Bath, South Building, Soldier Down Ln, Claverton Down, Bath BA2 7AY, U.K.; § STFC Rutherford Appleton Laboratory, Central Laser Facility, Didcot OX11 0FA, U.K.; ∥ Department of Meteorology, University of Reading, Whiteknights, Earley Gate, Reading RG6 6UR, U.K.

## Abstract

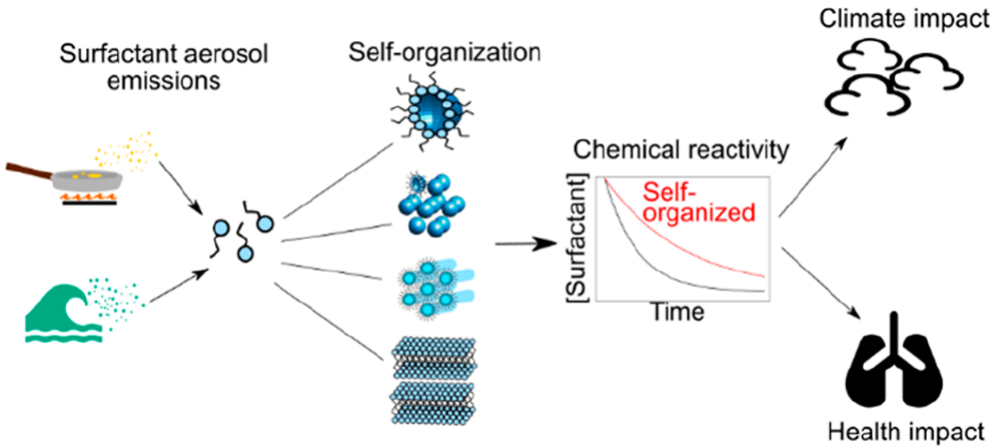

Aerosols are ubiquitous in the atmosphere. Outdoors, they take part in the climate system via cloud droplet formation, and they contribute to indoor and outdoor air pollution, impacting human health and man-made environmental change. In the indoor environment, aerosols are formed by common activities such as cooking and cleaning. People can spend up to *ca*. 90% of their time indoors, especially in the western world. Therefore, there is a need to understand how indoor aerosols are processed in addition to outdoor aerosols.

Surfactants make significant contributions to aerosol emissions, with sources ranging from cooking to sea spray. These molecules alter the cloud droplet formation potential by changing the surface tension of aqueous droplets and thus increasing their ability to grow. They can also coat solid surfaces such as windows (“window grime”) and dust particles. Such surface films are more important indoors due to the higher surface-to-volume ratio compared to the outdoor environment, increasing the likelihood of surface film–pollutant interactions.

A common cooking and marine emission, oleic acid, is known to self-organize into a range of 3-D nanostructures. These nanostructures are highly viscous and as such can impact the kinetics of aerosol and film aging (i.e., water uptake and oxidation). There is still a discrepancy between the longer atmospheric lifetime of oleic acid compared with laboratory experiment-based predictions.

We have created a body of experimental and modeling work focusing on the novel proposition of surfactant self-organization in the atmosphere. Self-organized proxies were studied as nanometer-to-micrometer films, levitated droplets, and bulk mixtures. This access to a wide range of geometries and scales has resulted in the following main conclusions: (i) an atmospherically abundant surfactant can self-organize into a range of viscous nanostructures in the presence of other compounds commonly encountered in atmospheric aerosols; (ii) surfactant self-organization significantly reduces the reactivity of the organic phase, increasing the chemical lifetime of these surfactant molecules and other particle constituents; (iii) while self-assembly was found over a wide range of conditions and compositions, the specific, observed nanostructure is highly sensitive to mixture composition; and (iv) a “crust” of product material forms on the surface of reacting particles and films, limiting the diffusion of reactive gases to the particle or film bulk and subsequent reactivity. These findings suggest that hazardous, reactive materials may be protected in aerosol matrixes underneath a highly viscous shell, thus extending the atmospheric residence times of otherwise short-lived species.

## Key References

- PfrangC.; RastogiK.; Cabrera-MartinezE. R.; SeddonA. M.; DickoC.; LabradorA.; PlivelicT. S.; CowiesonN.; SquiresA. M.Complex Three-Dimensional Self-Assembly in Proxies for Atmospheric Aerosols. Nat. Commun.2017, 8( (1), ), 172410.1038/s41467-017-01918-129170428PMC5701067.^1^*The first study using the levitation-SAXS-Raman technique to probe surfactant self-organization in an organic aerosol proxy and its effect on reaction kinetics.*
- MilsomA.; SquiresA. M.; WodenB.; TerrillN. J.; WardA. D.; PfrangC.The Persistence of a Proxy for Cooking Emissions in Megacities: A Kinetic Study of the Ozonolysis of Self-Assembled Films by Simultaneous Small and Wide Angle X-Ray Scattering (SAXS/WAXS) and Raman Microscopy. Faraday Discuss.2021, 226, 364–38110.1039/D0FD00088D33284926.^2^*A kinetic study presenting a novel method of measuring reaction kinetics using synchrotron X-ray scattering. We quantified the effect of molecular self-organization on oleic acid ozonolysis reaction kinetics and showed that there is both a thickness and a phase state dependence.*
- MilsomA.; SquiresA. M.; QuantI.; TerrillN. J.; HubandS.; WodenB.; Cabrera-MartinezE. R.; PfrangC.Exploring the Nanostructures Accessible to an Organic Surfactant Atmospheric Aerosol Proxy. J. Phys. Chem. A2022, 126, 733110.1021/acs.jpca.2c0461136169656PMC9574911.^3^*Systematic assessment of the self-organized nanostructures accessible to the oleic acid–sodium oleate proxy. The composition was controlled by adding commonly co-emitted compounds (stearic acid and sugars) as well as changing the amount of salinity of the aqueous phase.*
- MilsomA.; SquiresA. M.; WardA. D.; PfrangC.The Impact of Molecular Self-Organisation on the Atmospheric Fate of a Cooking Aerosol Proxy. Atmos. Chem. Phys.2022, 22( (7), ), 4895–490710.5194/acp-22-4895-2022.^4^*A kinetic multilayer modeling study demonstrating the impact that self-organized nanostructure formation has on the chemical lifetime of oleic acid. We show how molecular diffusivity evolves during ozonolysis and that the formation of a crust limits the rate of reaction.*

## 1 Introduction

Aerosols influence the climate, air quality, and human health.^5,6^ The climatic impact is either through the direct interaction of aerosols with the sun’s radiation or indirectly through the cloud droplets that are formed by them. Aerosols transport harmful pollutants through the atmosphere.^7^ These pollutants can be breathed in and have a negative impact on human health.^8,9^ People in the West spend *ca*. 90% of their time indoors,^10,11^ making indoor aerosols generated by processes such as cooking^12,13^ and cleaning^12^ important to consider from an air quality and health perspective. Particulate matter of less than 2.5 μm (PM_2.5_) is a major global public health risk, increasing the risk of mortality from diseases such as lung cancer.^14^ It is therefore important to understand what influences the chemical lifetime of aerosol components. This lifetime influences the climatic and human health effects of aerosols.^5,8,15^

Films made of deposited aerosol particles and condensed semivolatile species can form on indoor surfaces such as windows and furniture.^16,17^ Indoor surface chemistry is particularly important due to the higher surface-to-volume ratio indoors compared to in an urban environment, increasing the likelihood of film–pollutant interactions. Laboratory experiments on films deposited on solid substrates, such as those presented here,^2,3^ are needed to help us understand how these films are likely to age indoors.

Viscosity is a key determiner of the rate of uptake of trace gases to an aerosol particle.^18^ Two important aerosol processes, water uptake and oxidation, involve trace gas uptake. A range viscosities are possible for atmospheric aerosols,^19−21^ and highly viscous media reduce the diffusion coefficient of small molecules through a particle. This is illustrated by the characteristic time of mass transport and mixing, τ_d_, which is related to the particle diameter (*d*_p_) and the diffusion coefficient (*D*) of the molecule in question through eq 1.^18^
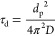
1τ_d_ can vary from a few seconds in the liquid phase to hours and days for the semisolid (i.e., with a viscosity of ∼10^2^–10^12^ Pa s)^18^ and solid phases. Highly viscous particles will therefore age much slower and take longer to form cloud droplets.

Surfactants have been characterized in atmospheric aerosols and influence cloud droplet formation through the depression of droplet surface tension, lowering the humidity required for water droplets to grow.^22^ A common surfactant emission is oleic acid. This unsaturated fatty acid is a major component of cooking emissions,^23,24^ which can make up ∼10% of PM_2.5_ in the U.K.^25^ Oleic acid is also emitted in the marine environment, where it has a biogenic source known as the sea surface microlayer.^26,27^

**Figure 1:**
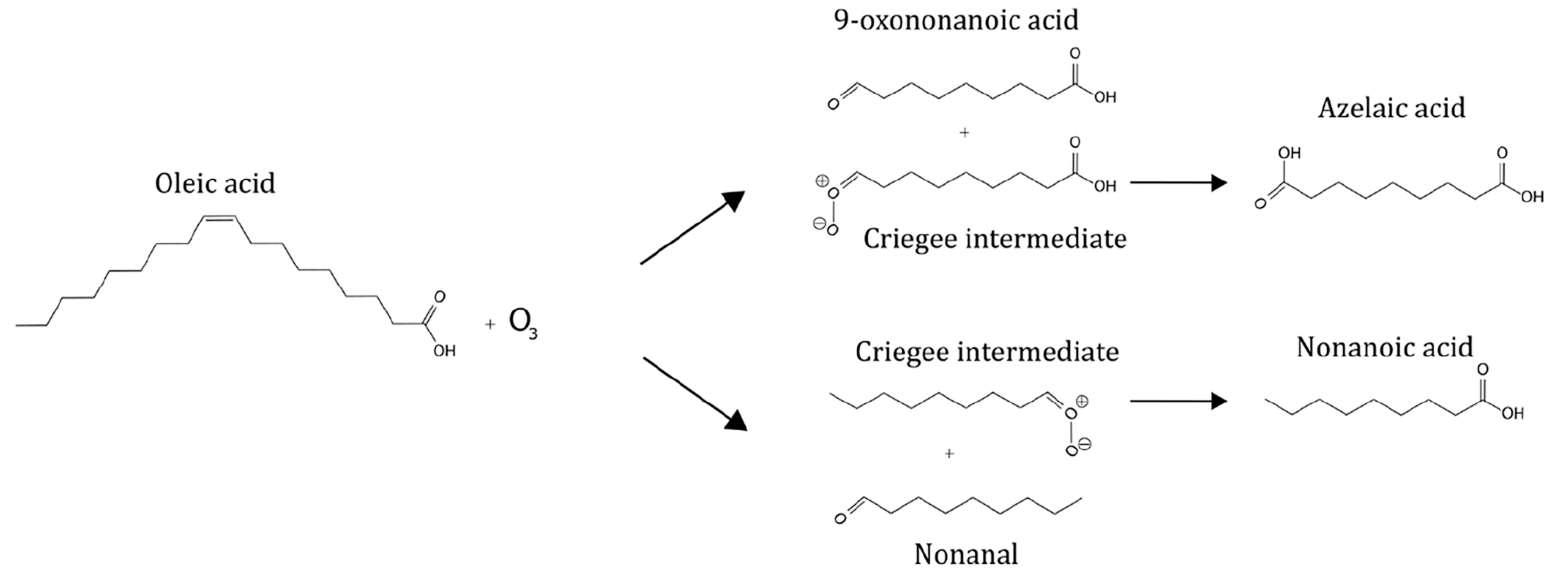
Oleic acid–ozone heterogeneous reaction scheme showing the principal products. Reproduced with permission from ref (32). Copyright 2021 the authors. Published by Copernicus Publications under a Creative Commons Attribution 4.0 International (CC BY 4.0) License.

The chemical lifetime of particle-bound compounds can have a direct effect on our health^8,15^ and influence particle hygroscopicity.^28^ It is therefore important to understand the kinetics of aerosol atmospheric aging. The oleic acid–ozone heterogeneous reaction is a model system for reactive organic aerosol in the atmosphere.^29−32^ Primary products include Criegee intermediates, nonanal, nonanoic acid, 9-oxononanoic acid, and azelaic acid (Figure 1). These products go on to form diperoxides, secondary ozonides, and higher-molecular-weight oligomers.^33^

There remains a discrepancy between the longer measured atmospheric lifetime of oleic acid and the chemical lifetime measured in the laboratory.^34−36^ Recent fieldwork has demonstrated that the *trans* form of oleic acid, elaidic acid, reacts 38 ± 5% slower with ozone than oleic acid.^36^ The authors suggested that the steric arrangement for elaidic acid would be similar to that for saturated fatty acids, which are known to crystallize to form solid or semisolid phases.^3^ The formation of viscous phases in aerosol particles may provide an explanation for this observation. The oleic acid–ozone reaction is therefore an ideal candidate for the studies we present here due to this discrepancy and the abundant literature for comparison.

**Figure 2:**
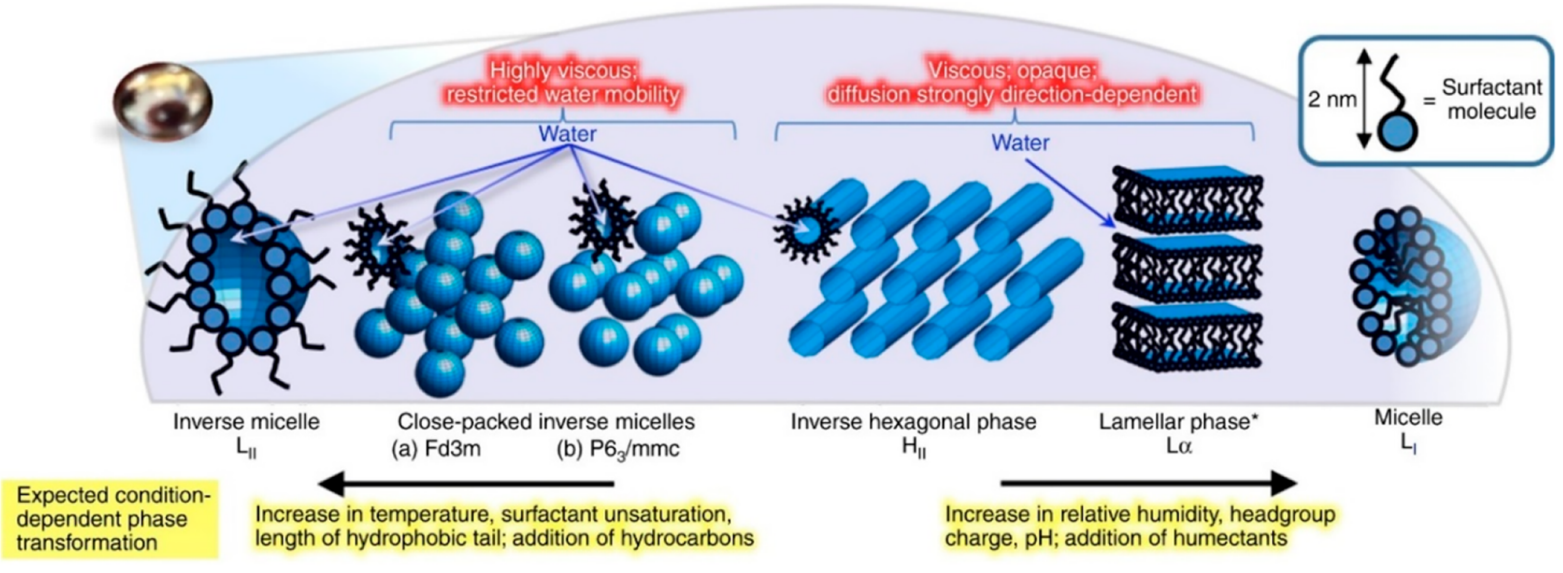
Complex 3D self-organization of surfactant molecules in proxies for atmospheric aerosols. *The lamellar phase can exist over a much wider range of relative humidities than the other phases. Reproduced with permission from ref (1). Copyright 2017 the authors. Published by Springer Nature under a Creative Commons Attribution 4.0 International (CC BY 4.0) License.

Surfactants possess hydrophilic heads and hydrophobic tails. Under conditions likely found in the atmosphere, oleic acid and its sodium salt can self-organize into a range of structures known as lyotropic liquid crystal (LLC) phases, illustrated in Figure 2.^1,37^ The LLC phases formed by the oleic acid–sodium oleate system are “inverse”, where the water is encapsulated within these structures, a so-called “water-in-oil” phase. These phases have atmospherically important properties (Table 1). The same molecule can exhibit significantly different physical properties dependent on its molecular arrangement. These unique structures and their properties have been exploited by soft-matter scientists in fields including drug delivery^38^ and templating electrode surfaces for catalysis.^39^

**Table 1:** Key Physical Properties of LLC Phases and Their Implications for Atmospheric Aerosols

Property	Implication
Viscosity	Some LLC phases are highly viscous and appear like toothpaste, while others flow more readily. LLC phase viscosity can vary across *ca.* 2–4 orders of magnitude^1,40,41^ so that mixing times of atmospherically relevant molecules would differ significantly between LLC phases (eq 1).
Directionally dependent diffusivity	Certain phases (e.g., the lamellar phase) exhibit directionally dependent diffusion. For lamellar phases, diffusion is generally faster parallel to the bilayers compared to perpendicular.^42^
Opacity	The inverse micellar phase is translucent whereas the inverse hexagonal phase is opaque. This means that the same molecule interacts with light differently depending on the self-organized structure it forms. This has a potential climatic impact.

Micelle formation has been considered in the atmospheric literature,^43^ and the concept of the critical micelle concentration (CMC) is well-known by those who model the thermodynamics of atmospheric surfactants.^44,45^ Prior to our work, there had not been any systematic study of the effect of LLC phase formation on atmospherically relevant aerosol processes.

We propose that surfactant molecular self-organization is a real possibility under atmospheric conditions. The work presented here showcases our recent endeavors into this novel proposition.

This Account will first introduce the techniques used to probe LLC phases. The results will then be described in terms of (i) phases identified and initial qualitative findings; (ii) quantitative kinetic and aging experiments on micro- and nanometer-scale films; (iii) observations of core–shell morphologies in aging levitated particles; and (iv) modeling the impact on the surfactant atmospheric chemical lifetime.

## 2 Techniques Used

### 2.1 Small-Angle X-ray Scattering (SAXS)

**Figure 3:**
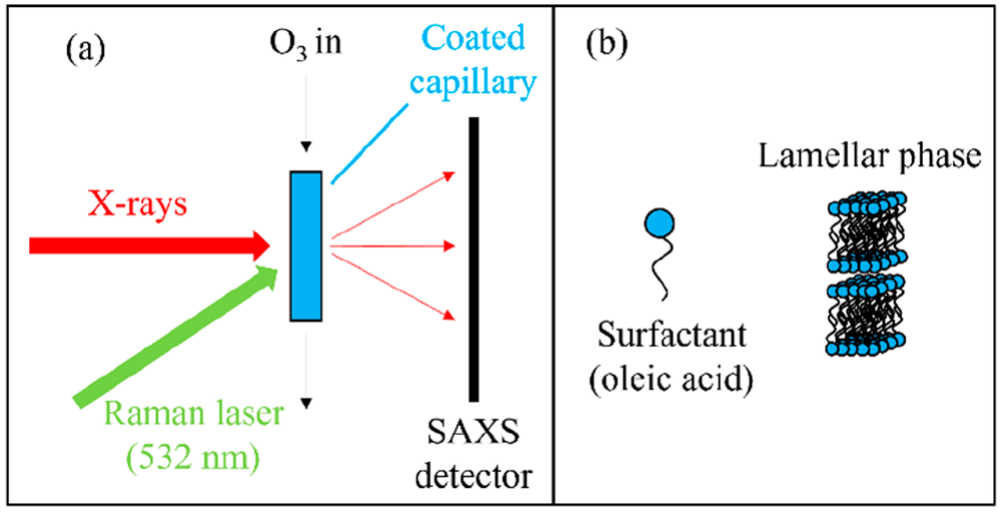
(a) A schematic representation of the small-angle X-ray scattering (SAXS) and Raman spectroscopy experiments. (b) The lamellar phase formed by oleic acid and sodium oleate. Reproduced with permission from ref (4). Copyright 2022 the authors. Published by Copernicus Publications under a Creative Commons Attribution 4.0 International (CC BY 4.0) License.

Techniques used to probe surfactant self-organization on the nanometer scale are small- and wide-angle X-ray scattering (SAXS and WAXS) (Figure 3).^46^ The LLC and dry crystalline phases that we studied return characteristic Bragg peaks in the SAXS pattern, allowing us to identify the specific nanostructures. WAXS can be used to probe the shorter length scales associated with closely packed, well-ordered alkyl chains in the case of the oleic acid–sodium oleate proxy.^47^

The distances between repeating units (e.g., between lamellar bilayers) can be derived through the calculation of the *d* spacing for a particular scattering peak (eq 2).
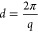
2The *d* spacing is a function of the X-ray wavelength (λ) and momentum transfer (*q*), which is related to the scattering angle (θ) (eq 3).
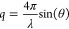
3SAXS and WAXS can allow us to track changes in repeat distances in both coated capillaries and levitated particles, which we took advantage of to draw atmospheric implications.^1−3,47^

### 2.2 Acoustic Levitation with Simultaneous Raman Spectroscopy and SAXS/WAXS

**Figure 4:**
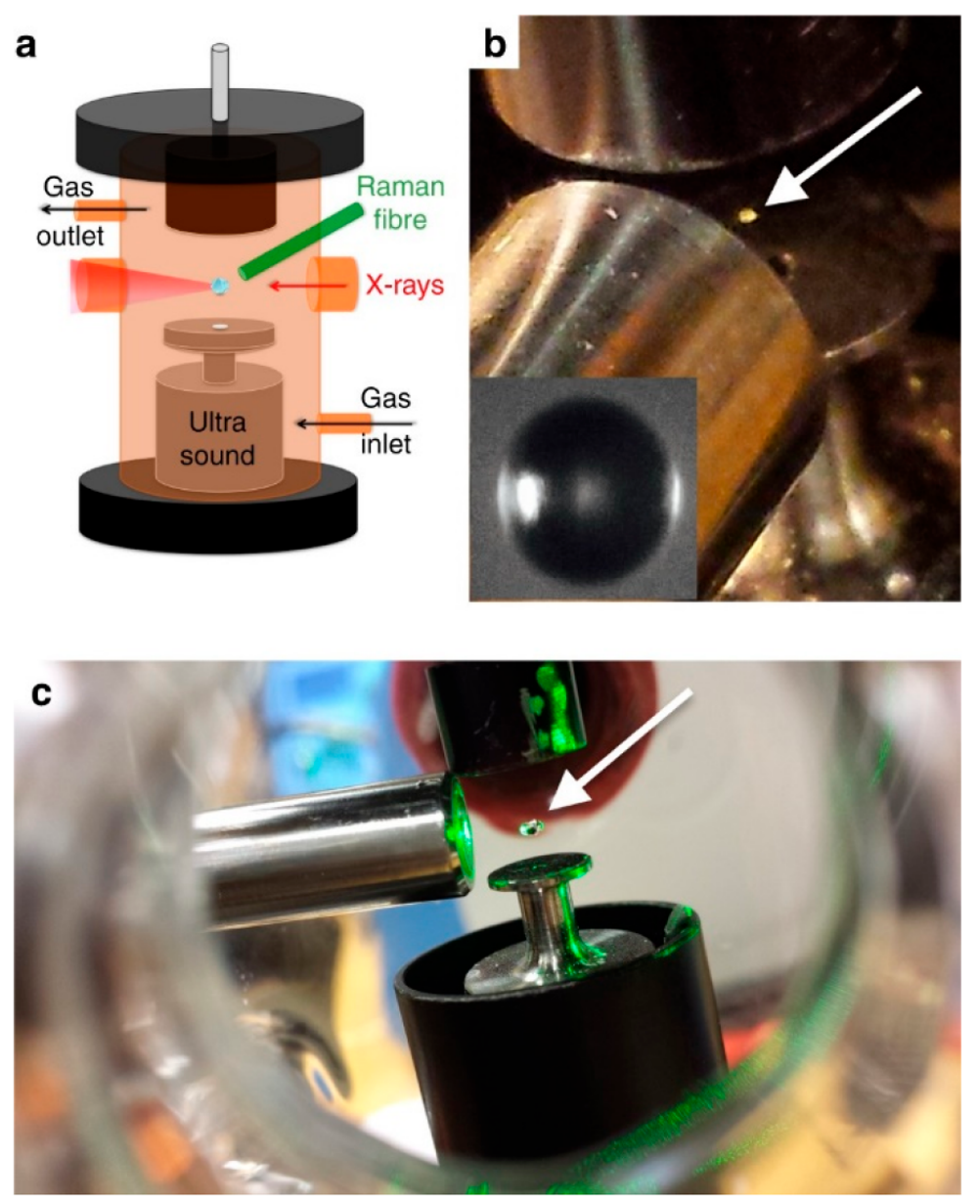
(a) Schematic diagram of the simultaneous Raman-acoustic levitation system. (b) Photograph of the online setup with a Raman probe and a levitated 80 μm droplet (inlay shows the microscopic image of an 80-μm droplet). (c) Photograph of offline setup with a 532 nm laser illuminating the droplet. Droplet locations are highlighted by white arrows. Reproduced with permission from ref (1). Copyright 2017 the authors. Published by Springer Nature under a Creative Commons Attribution 4.0 International (CC BY 4.0) License.

A key technical development from our work has been to combine acoustic levitation with SAXS/WAXS and Raman spectroscopy (Figure 4).^1,3,37,47^ The gas-phase environment of the levitated particle can be controlled, enabling the exposure of single levitated particles to changes in humidity and gaseous oxidants (e.g., ozone).

Levitation–Raman–SAXS allows the simultaneous measurement of both the chemical and structural features of a levitated particle. We are therefore able to link chemical kinetics with structural changes happening in a levitated particle of the oleic acid–sodium oleate proxy. One can distinguish structural differences between the core and shell of a levitated particle,^37^ and the microfocus capability of the I22 beamline at the Diamond Light Source (U.K.) has enabled high spatial resolution (section 4).^47,48^

### 2.3 Neutron Reflectometry (NR) and Grazing Incidence SAXS (GI-SAXS) on Spin-Coated Films

**Figure 5:**
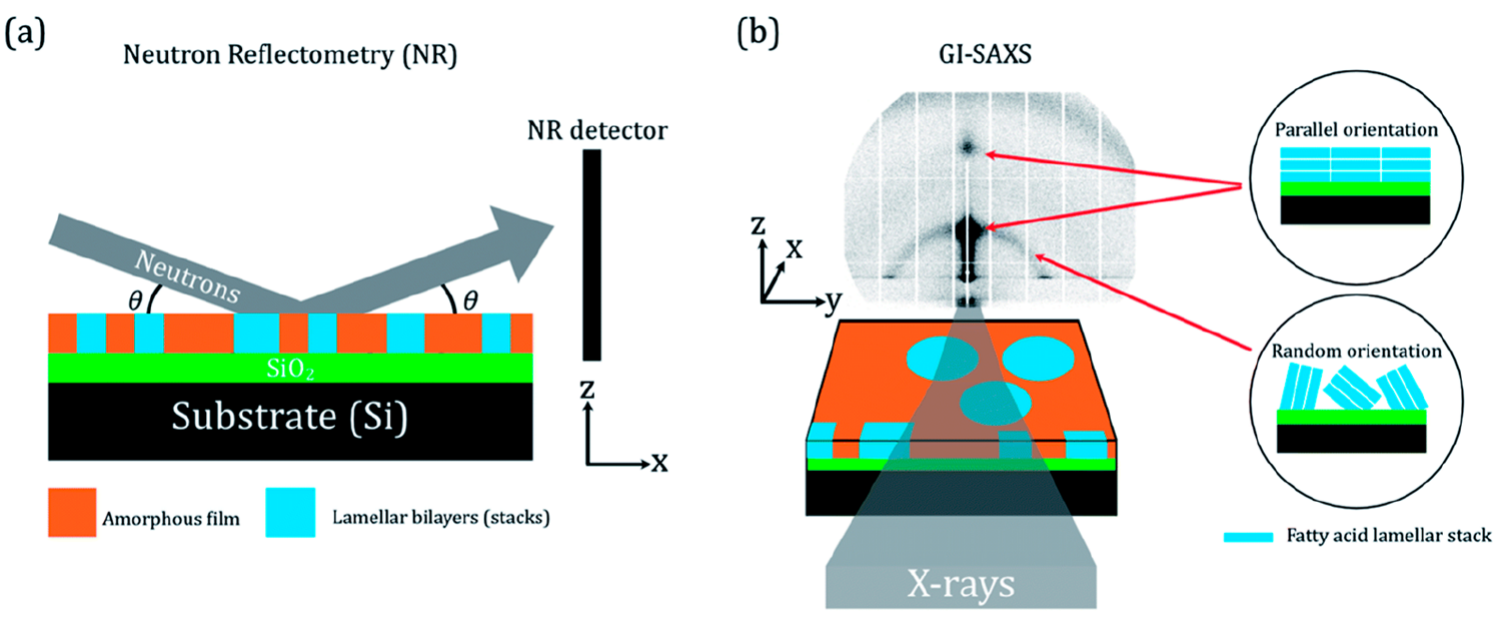
Schematic representations of (a) neutron reflectometry (NR) and (b) grazing-incidence small-angle X-ray scattering (GI-SAXS) experiments. The GI-SAXS data presented are from a film coated on a silicon wafer at 2000 rpm. The mixed area model is illustrated, showing regions of the amorphous film and lamellar bilayers (stacks). The relationship between the lamellar stack orientation and scattering pattern is illustrated in (b). The X-rays and neutrons travel along the *x* axis in the positive direction, the *x*–*z* plane is the specular plane, and the angle of incidence (θ) is identified in panel (a). Reproduced with permission from ref (59). Copyright 2022 the authors. Published by Royal Society of Chemistry under a Creative Commons Attribution 3.0 Unported (CC BY 3.0) License.

Neutron reflectometry (NR) is a technique used to derive a depth-resolved profile of a thin film, returning properties such as the film thickness and roughness.^49^ The principle of NR is similar to that of SAXS: a neutron beam hits a sample at low incident angles and is reflected at the interface between layered structures present in the sample (Figure 5(a)). An advantage of NR is the ability to use contrast variation with selective deuteration of the molecules of interest to resolve mechanistic details. If the sample is deuterated, then there would be a larger contrast in the scattering ability, or scattering length density (SLD), of the sample compared with that of adjacent layers (i.e., air and substrate). This allows us to see interference fringes clearly in the NR curve, which arise from this SLD contrast.

The fraction of incident neutrons specularly reflected, the reflectivity (*R*), is related to *q* and the SLD:
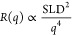
4In grazing incidence-SAXS (GI-SAXS), X-rays illuminate the sample at small “grazing” incident angles. If the sample has self-organized phases present, then the orientation of those structures can be determined (Figure 5(b)).

### 2.4 Kinetic Multilayer Modeling

**Figure 6:**
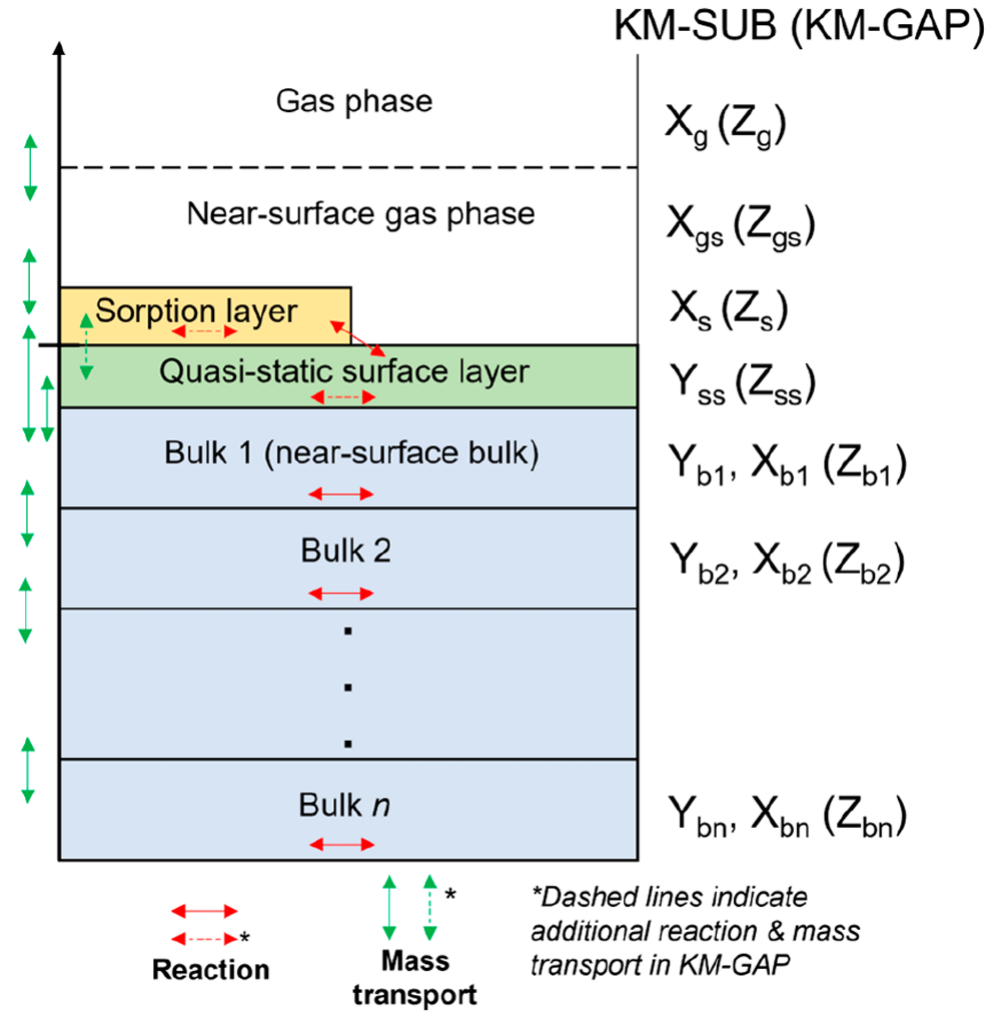
Schematic representation of a kinetic multilayer model of an aerosol or film. Reproduced with permission from ref (58). Copyright 2022 the authors. Published by Copernicus Publications under a Creative Commons Attribution 4.0 International (CC BY 4.0) License.

State-of-the-art kinetic multilayer modeling has been used to describe physical and chemical processes in both aerosol particles and films.^50−52^ These models are detailed and explicitly treat the adsorption and desorption of volatile molecules (e.g., O_3_, NO_2_, water vapor, etc.), surface and bulk reactions, and mass transport through the particle or film bulk (Figure 6). Optimization of these detailed models gives us access to key physical parameters (e.g., diffusion and reaction rate coefficients) which would otherwise be difficult or impossible to determine experimentally. Kinetic multilayer models can be used to assess the impact of environmental changes on the persistence of chemicals incorporated in the particle or film.^4,15^

Recent developments in multilayer modeling include the incorporation of film growth mechanisms,^53^ the treatment of the lung epithelial lining fluid to derive health implications from these models,^54,55^ an educational tool,^56^ and a move toward machine learning algorithms.^57^ We have recently published open-source software, MultilayerPy, which facilitates the creation and optimization of these models.^58^ MultilayerPy is free for anyone to use and has the potential to incorporate current and future multilayer models.

## 3 Qualitative Indications of the Atmospheric Importance of LLC Formation

We looked qualitatively at LLC phases accessible to the oleic acid–sodium oleate–brine system and its resistance to chemical aging.^1^ Humidity change experiments revealed the range of phases observed in levitated particles of this proxy, summarized in Figure 2. Generally, increasing the humidity resulted in LLC phases with larger water-to-surfactant ratios.

**Figure 7:**
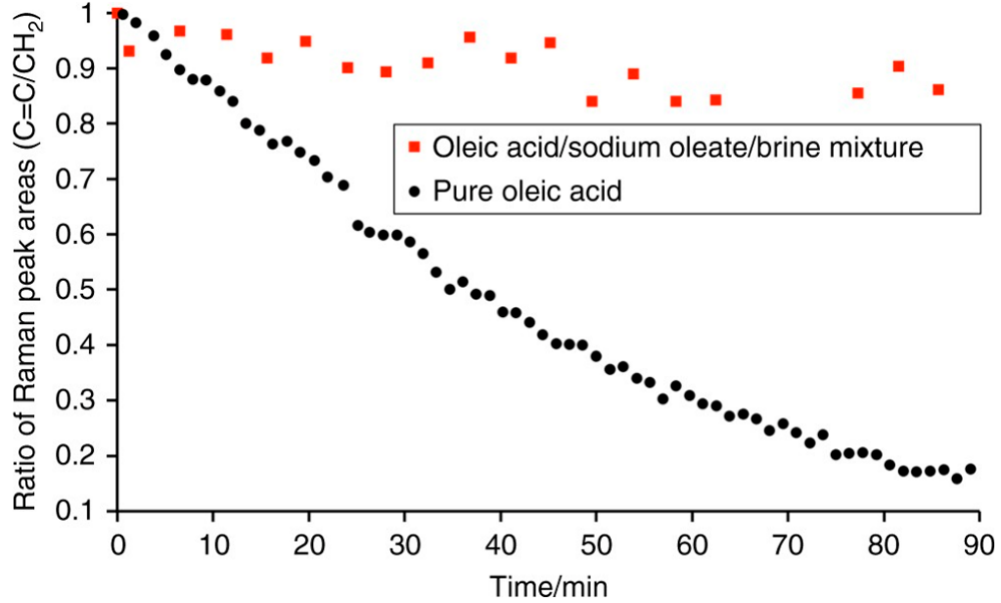
Ozonolysis of self-assembled mixture vs pure oleic acid. Pure, liquid oleic acid droplets (∼200 μm diameter) as well as droplets of an oleic acid/sodium oleate/brine mixture (∼195 μm diameter). The droplets were exposed to the same ozone-mixing ratio of ∼28 ppm. Reproduced with permission from ref (1). Copyright 2017 the authors. Published by Springer Nature under a Creative Commons Attribution 4.0 International (CC BY 4.0) License.

Self-organization was destroyed when these particles were exposed to ozone. The kinetics of this reaction were much slower for the self-organized phase compared with pure liquid oleic acid (Figure 7).^1^ This confirmed our assumption that these viscous self-organized LLC phases would slow heterogeneous oxidation due to reduced oleic acid and ozone diffusivity. This observation justified our more quantitative kinetic work described in section 3.

We carried out a systematic study of the phase-composition relationship for the oleic acid–sodium oleate system in both bulk mixtures and levitated droplets.^3^ The addition of compounds commonly co-emitted with oleic acid such as sugars (glucose, fructose, and sucrose) and a saturated fatty acid (stearic acid) altered the observed LLC phase.^1,3^ In some instances, a difference in the sugar added meant the difference between an inverse hexagonal phase (opaque and directionally dependent diffusion) and a close-packed inverse micellar phase (viscous and translucent). More complex mixtures informed by atmospheric measurements and with up to 6 components mostly returned the inverse hexagonal and close-packed inverse micellar LLC phases. Small changes in NaCl concentration caused LLC phase transitions in addition to coexisting phases and phase separation, observed visually.

**Figure 8:**
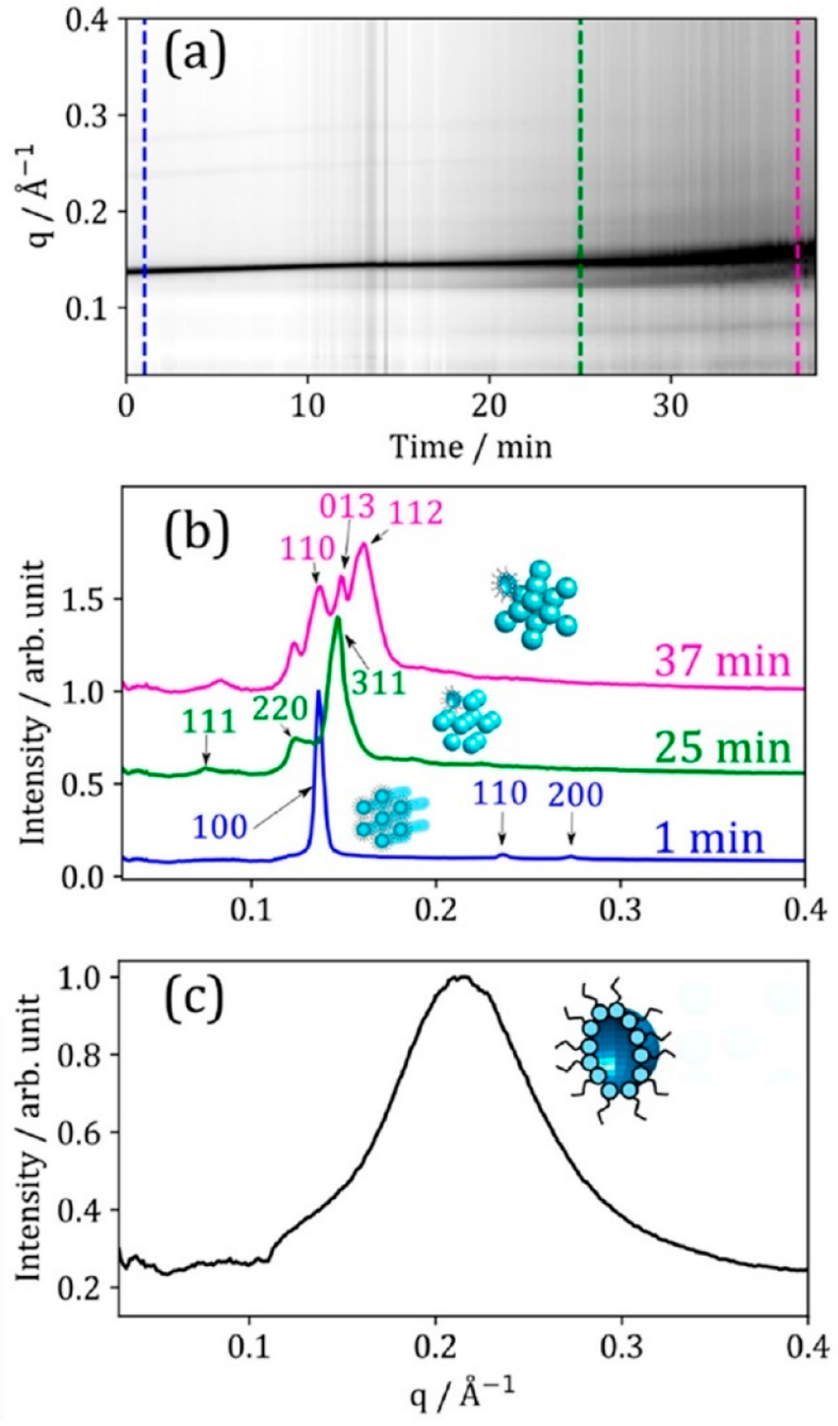
(a) One-dimensional SAXS pattern vs time during dehumidification from ∼86% to ∼12% RH. Colored dashed lines correspond to SAXS patterns in panel (b). (b) Selected 1D SAXS patterns from the same experiment. The key Miller (hkl) indices for each phase, along with a cartoon of each phase, are labeled: inverse hexagonal (1 min); cubic close-packed inverse micelles (Fd3m); and hexagonal close-packed inverse micelles (P63/mmc). (c) One-dimensional SAXS pattern from the center of the droplet after rehumidification from ∼12% to ∼83% RH. Reproduced with permission from ref (3). Copyright 2022 the authors. Published by the American Chemical Society under a Creative Commons Attribution 4.0 International (CC BY 4.0) License.

The trends observed in bulk mixtures were reproduced in levitated droplets (Figure 8). Dehumidification of a mixture of oleic acid–sodium oleate in a NaCl solution followed the expected trend when decreasing the amount of water and increasing the salt concentration: dryer, saltier mixtures favor LLC phases with increased surfactant–water interfacial curvature (i.e., inverse micelles have a greater interfacial curvature than the cylindrical structures in the initial inverse hexagonal phase).^3^ Aerosols can undergo rapid changes in their environmental humidity (i.e., entering a cloud vs leaving a cloud). These results show that any potential surfactant LLC phase in an aerosol could switch between phases which vary significantly in viscosity,^40^ with implications for bulk-phase mixing times.^18^

**Figure 9:**
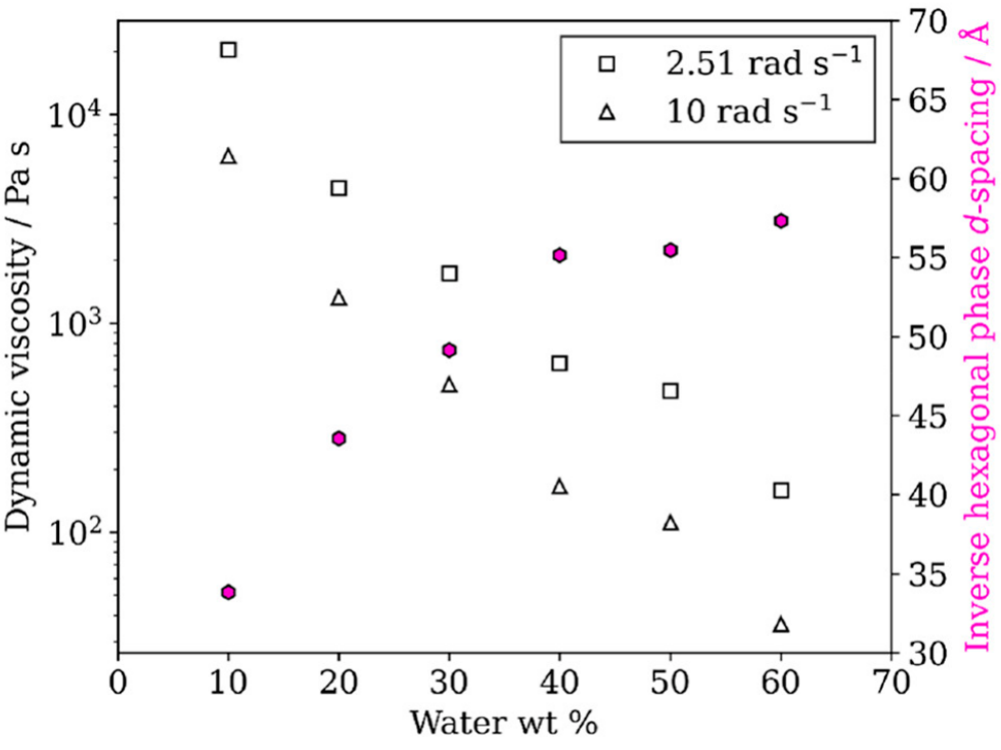
Dynamic viscosity of a 1:1 oleic acid–sodium oleate mixture vs water content (wt % water) at two different oscillatory frequencies together with corresponding *d* spacing for the dominant inverse hexagonal phase. Reproduced with permission from ref (3). Copyright 2022 the authors. Published by American Chemical Society under a Creative Commons Attribution 4.0 International (CC BY 4.0) License.

We quantified the dynamic viscosity of a representative self-organized mixture for a range of water contents and linked it to the size of the water channels measured, related to the *d* spacing (Figure 9).^3^ The values of ∼10^2^–10^4^ Pa s are consistent with a semisolid state^18^ and quantify the effect of self-organization on mixture viscosity.

## 4 Studies on Micro- and Nanometer-Scale Films

Having qualitatively shown that surfactant self-organization is possible, has a measurable impact on viscosity, and slows down oxidation kinetics, we now present quantitative kinetic and hygroscopicity work on these self-organized systems as deposited films as proxies for aerosol coatings and indoor films.

**Figure 10:**
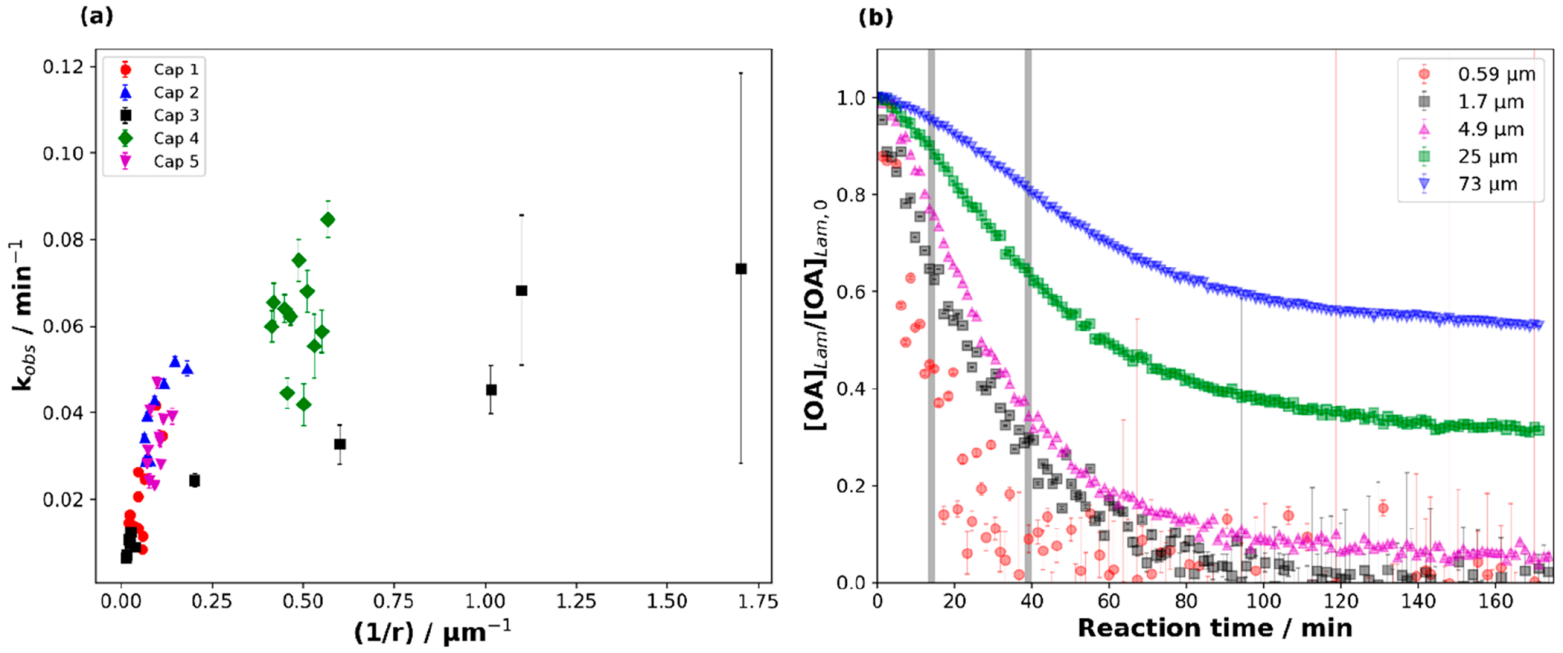
(a) The observed pseudo-first order decay constant (*k*_obs_) for the ozonolysis of oleic acid vs inverse film thickness (1/*r*). Different capillary (“Cap”) experiments are distinguished by different colors/symbols. (b) Decay plots of the normalized amount of lamellar oleic acid ([OA]_Lam_/[OA]_Lam,0_) vs reaction time (*t*) for different thicknesses (data are from one capillary experiment (“Cap 3”); gray bars indicate reaction times between which *k*_obs_ was measured for films with *r* > 2.5 μm). [O_3_] is 77 ± 5 ppm. Reproduced with permission from ref (2). Copyright 2020 the authors. Published by Royal Society of Chemistry under a Creative Commons Attribution 3.0 Unported (CC BY 3.0) License.

We developed a novel high-throughput method of following the reaction kinetics of the oleic acid–sodium oleate films reacting with ozone using SAXS data.^2^ In this case, the fatty acid was in the lamellar form and was coated as a film inside quartz capillaries, depicted in Figure 3. The synchrotron beamline we used^48^ enabled us to follow structural changes at various positions along the coated film in the same capillary. Taking advantage of the nonuniform film thickness along the capillary, we were able to follow the reaction kinetics for films of different thicknesses under exactly the same conditions (Figure 10).

There was a clear thickness dependence of the observed reaction rate (*k*_obs_) calculated from the decay in the lamellar phase scattering peak during ozone exposure. Thicker films reacted slower than thinner films (Figure 10(a)). A surface crust of product material may have formed during the reaction, explaining the apparent slowing down and even stopping of the reaction for films ≥5 μm (Figure 10(b)).

**Figure 11:**
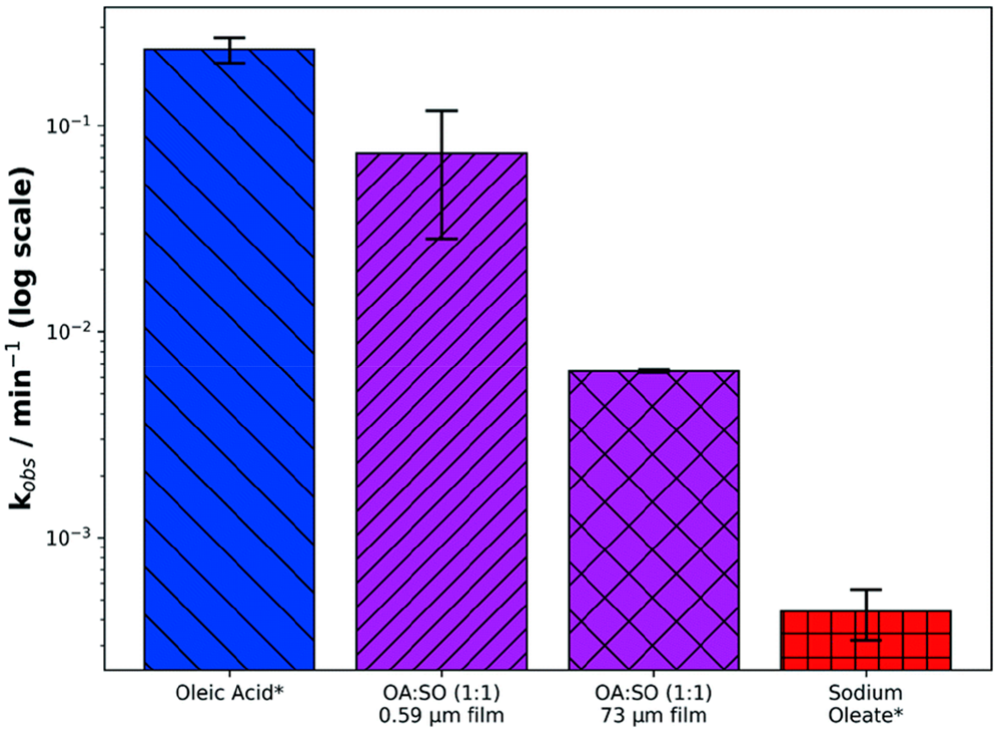
Comparison of *k*_obs_ among a capillary coating of oleic acid (liquid), two coatings of self-assembled oleic acid–sodium oleate proxy (semisolid: 0.59 and 73 μm thick), and a coating of sodium oleate (solid). *Oleic acid and sodium oleate decays were followed by Raman microscopy, using the C=C band at 1650 cm^–1^. The thickness of oleic acid and sodium oleate coatings was ∼50 μm (OA: oleic acid; SO: sodium oleate). [O_3_] = 77 ± 5 ppm. Reproduced with permission from ref (2). Copyright 2020 the authors. Published by Royal Society of Chemistry under a Creative Commons Attribution 3.0 Unported (CC BY 3.0) License.

The difference in reactivity going from the liquid (pure oleic acid) to semisolid (lamellar) and solid (sodium oleate) phase states was quantified for oleic acid using this technique (Figure 11). There was roughly an order of magnitude difference in *k*_obs_ for each step among the liquid, semisolid, and solid forms of oleic acid.

Spin-coating allowed us to control the deposited film thickness down to the nanometer scale. Films of the oleic acid–sodium oleate proxy were coated with thicknesses of ∼24–51 nm.^59^ We found that these films were patchy, with some regions consisting of lamellar bilayers and others amorphous (i.e., no self-assembled structure) (Figure 5).

**Figure 12:**
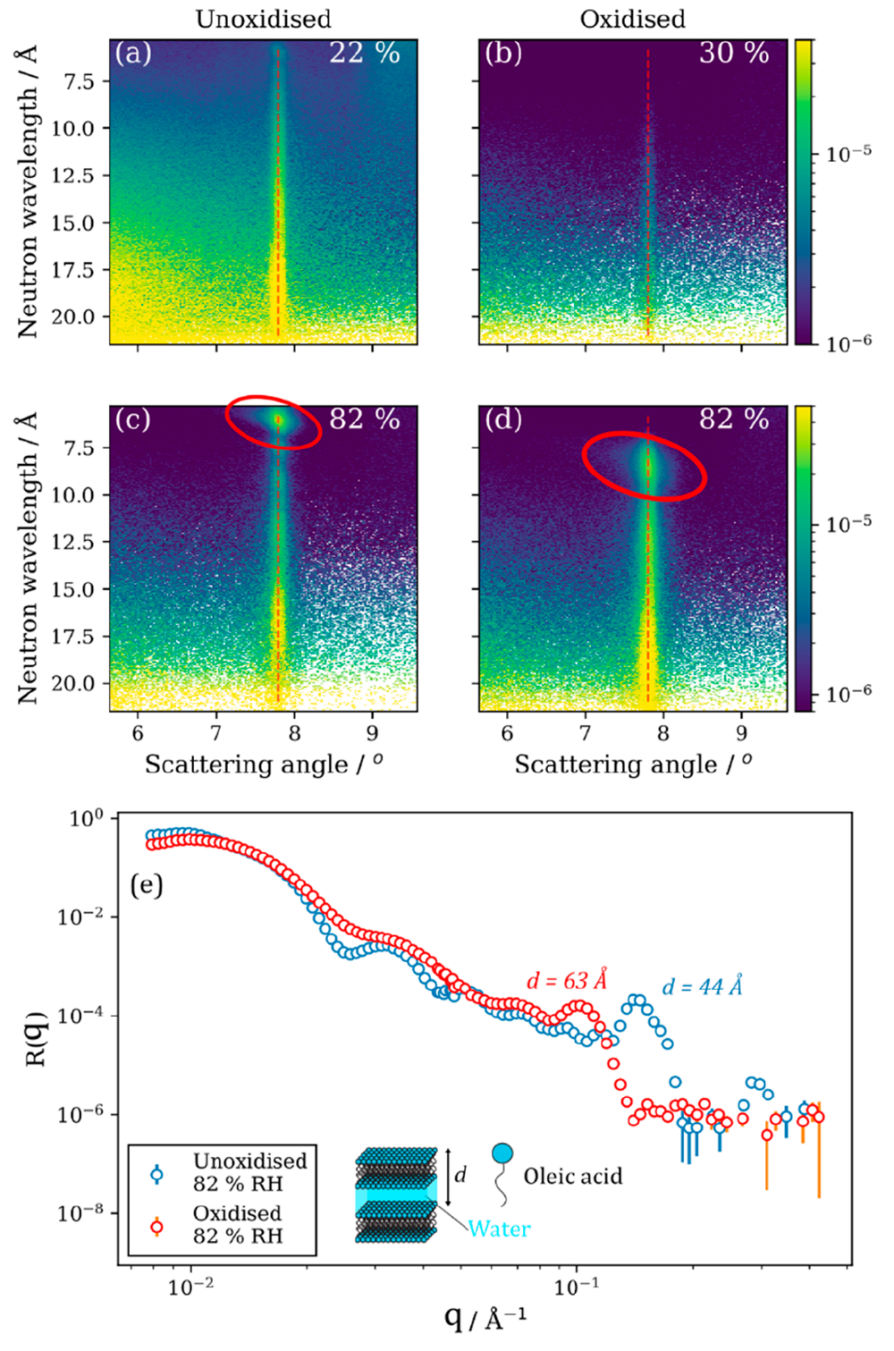
Off-specular NR measurements on (a and c) unoxidized and (b and d) oxidized films at low and high humidity (see top right of each panel for exact RH). The specular direction is denoted by the dashed red line, and the specular Bragg peak is highlighted by red circles in panels (c) and (d). (e) Comparison of 1-D specular NR curves for oxidized and unoxidized films at 82% RH. A schematic of the lamellar bilayer is presented along with the *d* spacings derived from the Bragg peak position. Reproduced with permission from ref (59). Copyright 2022 the authors. Published by Royal Society of Chemistry under a Creative Commons Attribution 3.0 Unported (CC BY 3.0) License.

A combination of GI-SAXS and NR revealed that the orientation of the lamellar bilayers was sensitive to humidity. Higher humidity induced orientation parallel to the substrate (Figures 5 and 12). This has implications for the uptake of small molecules through this lamellar region due to the directionally dependent diffusivity of molecules through this phase (Table 1). If most lamellar bilayers are oriented parallel to the substrate, then the diffusion of small molecules (e.g., ozone and water) through the film would be significantly reduced.^42^

We took advantage of the sensitivity of NR to deuterated molecules by exposing these nanometer-scale films to elevated humidity using D_2_O. This returned a clear signal for the parallel lamellar bilayer Bragg peak in the NR pattern in Figure 12(c) and (d), where the highlighted specular Bragg peak is evident upon humidification with D_2_O.

Further analysis of the specular NR pattern allowed us to compare the measured *d* spacing—the distance between the top of a bilayer and the top of the next bilayer, associated with the size of the bilayer and the amount of water in-between bilayers (Figure 12(e)). There was an ∼11-fold increase in the amount of water taken up by the lamellar bilayers when the deposited film was oxidized, compared to that taken up by unoxidized films. This demonstrates the importance of the oxidation state in influencing aerosol and film hygroscopicity.

## 5 Core–Shell Morphologies in Aging Particles

After hypothesizing a surface crust in section 3, we performed experiments investigating the spatial distribution of these self-organized phases during simulated atmospheric aging.

The small (∼16 μm × 12 μm) X-ray beam enabled us to take SAXS-WAXS patterns across an acoustically levitated droplet, allowing for the study of core–shell features.

**Figure 13:**
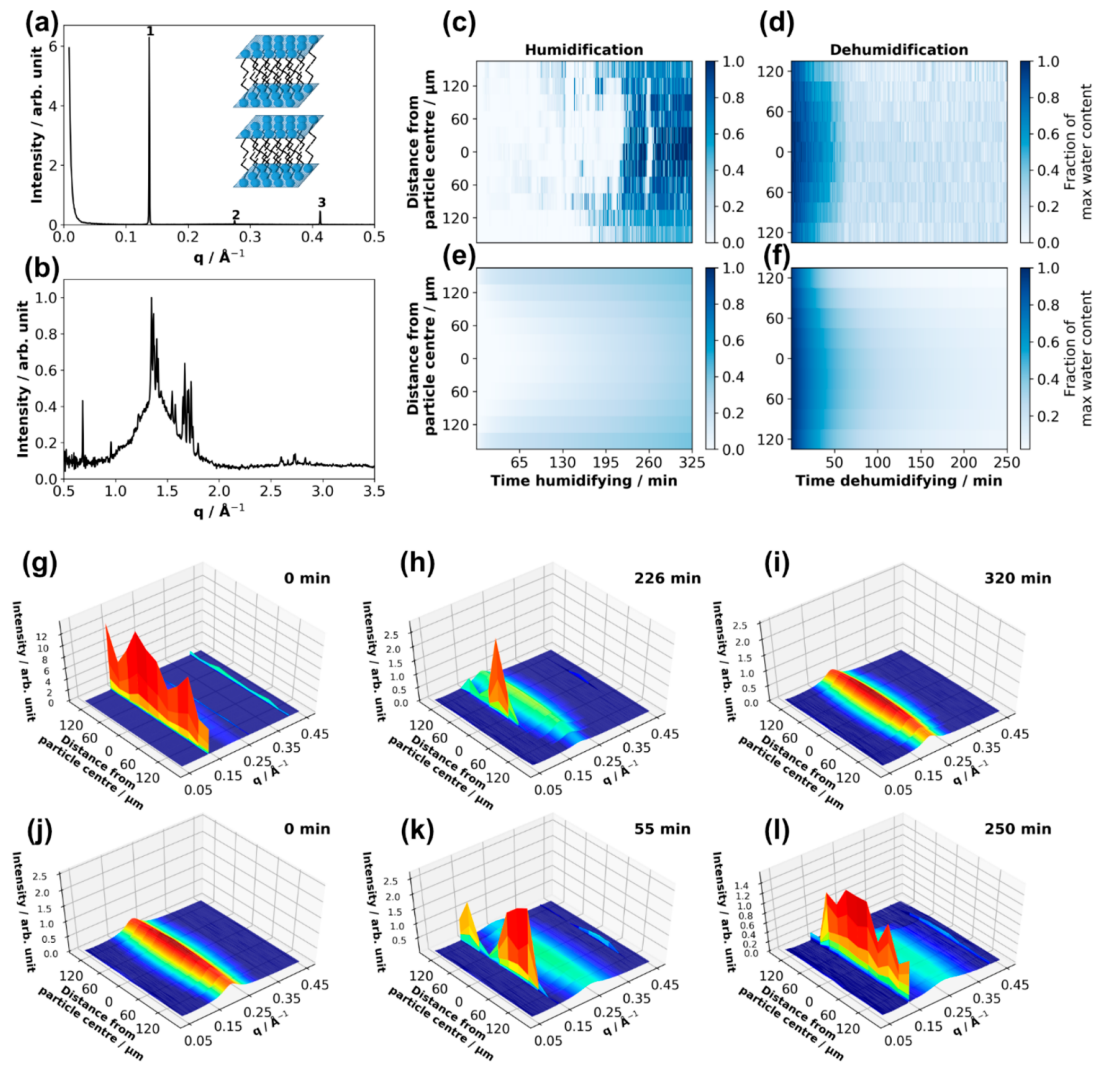
(a, b) SAXS and WAXS patterns obtained from a dry levitated particle of the acid–soap complex, with labeled lamellar peaks. (c, d) Experimental fraction of maximum water content as a function of distance from the particle center and time humidifying and dehumidifying. (e, f) Modeled fraction of maximum water. 3-D plots of 1-D SAXS patterns plotted against distance from the particle center for the same particle humidifying (g–i) and dehumidifying (j–l), with time humidifying and dehumidifying presented at the top right of each plot (particle size ≈ 150 μm (vertical radius) × 500 μm (horizontal radius). Humidification experiment: ∼38% (room RH) (g) to 90% RH (h, i); dehumidification experiment: 90% (j) to ∼38% RH (k, l). Reproduced with permission from ref (47). Copyright 2021 the authors. Published by Copernicus Publications under a Creative Commons Attribution 4.0 International (CC BY 4.0) License.

We identified a crystalline lamellar form of the oleic acid–sodium oleate system (Figure 13(a) and (b)) and levitated it in our acoustic trap.^47^ Vertical scans were taken of the levitated particle during humidification to ∼90% RH and observed changes in the self-organized structure.

A crystalline core–liquid crystalline–shell morphology was observed during humidification. The sharp peak for the crystalline lamellar phase at ∼0.14 Å^–1^ in the SAXS pattern dominated in the particle center, whereas the broad inverse micellar peak at ∼0.2 Å^–1^ dominated the outer shell of the particle (Figure 13(h)). This core–shell morphology was observed when reversing the humidity change from ∼90% to ∼38% RH (Figure 13(k)).

Core–shell effects are likely to impact the aging of atmospheric aerosols. We have already demonstrated the effect of the phase state on the oxidation of oleic acid by ozone (Figure 11).^2^ The crystalline (solid) form of oleic acid is much more viscous than the liquid crystalline inverse micellar phase formed in the shell during humidification. This implies a heterogeneity in the diffusivity of small molecules through the particle. We demonstrated this by fitting a simple multilayer model of water uptake and loss to our experimental findings (Figure 13(c–f)). Water diffusivity was estimated to be roughly an order of magnitude slower in the crystalline lamellar phase than in the liquid crystalline inverse micellar phase.^47^

**Figure 14:**
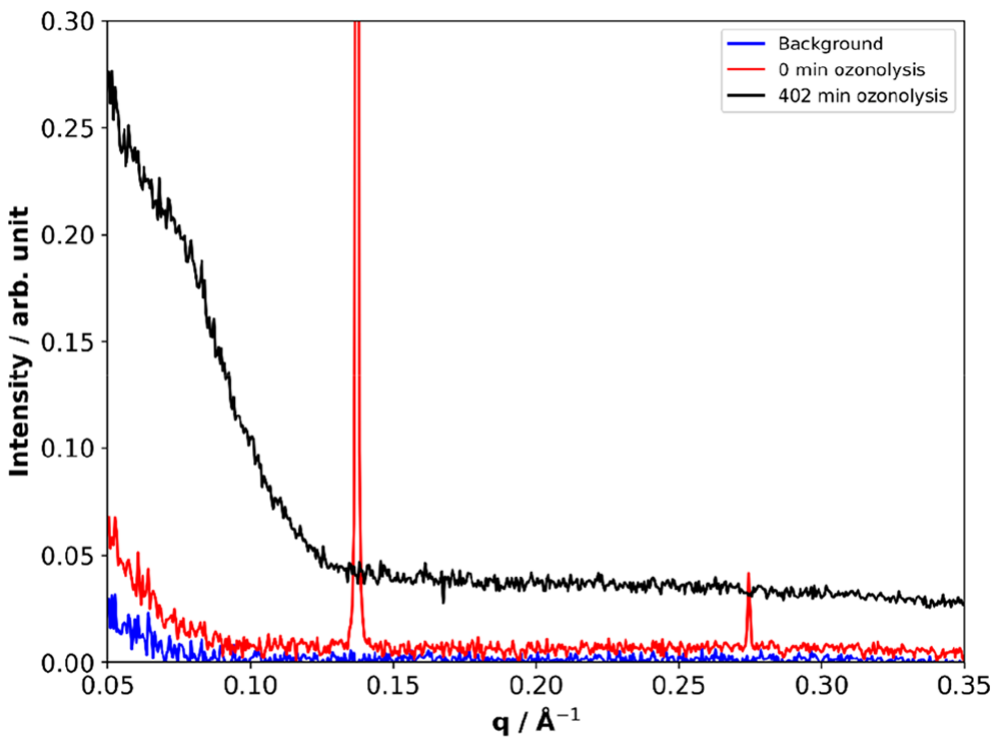
SAXS patterns of levitated acid–soap complex before and after ozonolysis compared with an empty-levitator background. There is a clear increase in low-*q* scattering due to ozonolysis. [O_3_] = 52 ± 0.5 ppm. Reproduced with permission from ref (47). Copyright 2021 the authors. Published by Copernicus Publications under a Creative Commons Attribution 4.0 International (CC BY 4.0) License.

Oxidative aging results in product aggregation at the surface of levitated particles.^47^ We showed this by studying the low-*q* region of the SAXS pattern at the surface of an aging crystalline lamellar oleic acid–sodium oleate mixture (Figure 14). From eq 2, we know that *q* is inversely proportional to the characteristic spacing, *d*, between aggregates. Therefore, scattering observed at low-*q* suggests aggregation on a longer length scale than the initial crystalline lamellar phase peaks. This is the first evidence of reaction product aggregation at the surface of an aging atmospheric aerosol particle proxy, to our knowledge. Surface crust formation has been hypothesized before in experimental and modeling studies^2,4,60,61^ and is thought to pose a diffusive barrier, limiting the reaction.

Simultaneous Raman spectroscopy showed that a significant fraction of the original carbon–carbon double bonds (34 ± 8%) remained in the levitated particle after 402 min of ozonolysis.^47^ We hypothesize that the now disordered oleic acid was still in a viscous medium, formed in part by the product aggregate, explaining why the reaction did not speed up after the initial crystalline lamellar phase was destroyed. Without the simultaneous Raman data, we would have assumed that all of the carbon–carbon double bonds had reacted due to the absence of the original SAXS pattern by the end of the experiment. This demonstrates the utility of combining SAXS and Raman spectroscopy.

## 6 Modeling the Atmospheric Chemical Lifetime Implications of Self-Organization

The following modeling work links the quantitative and qualitative experimental work described in sections 2, 3, and 4, combining kinetics with our experimental observations of a surface crust.

**Figure 15:**
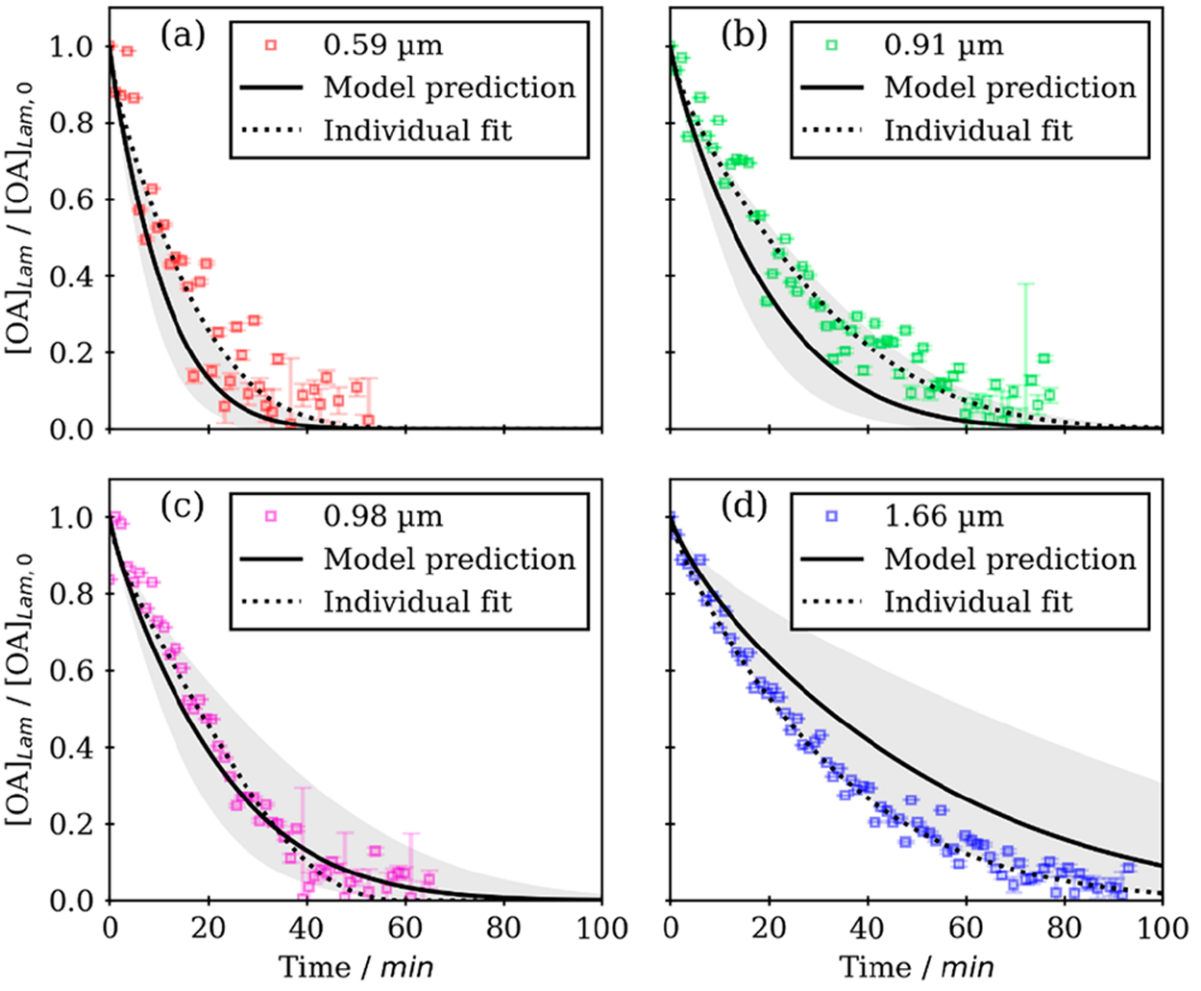
Kinetic decay plots of normalized lamellar-phase oleic acid concentration ([OA]_Lam_/[OA]_Lam,0_) vs time (experimental data from ref (12)); model predictions are based on optimized model parameters determined by fitting all data simultaneously. Individual fits to each data set are also presented. Film thicknesses are displayed in each legend. The gray-shaded regions represent the range of model outputs using parameter sets optimized from each individual fit. Reproduced with permission from ref (4). Copyright 2022 the authors. Published by Copernicus Publications under a Creative Commons Attribution 4.0 International (CC BY 4.0) License.

We developed a kinetic multilayer model of an aerosol surface and a bulk chemistry (KM-SUB)^51^ description of the ozonolysis of lamellar phase oleic acid films described in section 3.^4^ Input parameters associated with reaction rate constants and gas-surface-bulk exchange were set to literature-derived values. We were interested in the effect of the increased bulk viscosity, so the bulk diffusion coefficients of the reactants and products were varied using a global optimization algorithm. This optimization was carried out on individual decays and for all decays simultaneously, with uncertainty derived from the range of optimized parameters obtained from each individual fit (Figure 15). The model predictions in Figure 15 are from the model optimized to all four data sets simultaneously, representing the “average” best fit to all data sets.

Our optimized model reproduced our experimental observation that forming the lamellar phase resulted in an ∼1 order of magnitude decrease in the oleic acid half-life compared to that of the liquid form of oleic acid (Table 2).

**Table 2:** Approximate Half-Life of the Films Studied Taken from Individual Model Fits^a^

Film thickness/μm	Half-life/min
0.59 (Lam.)	∼11
0.91–1.66 (Lam.)	∼18–22
0.6–0.9^b^ (Liq.)	∼1–2

a Reproduced with permission from ref (4). Copyright 2022 the authors. Published by Copernicus Publications under a Creative Commons Attribution 4.0 International (CC BY 4.0) License.

b The range of half-lives from model outputs presented in Figure 12 (Lam.: lamellar-phase oleic acid; Liq.: liquid oleic acid).

**Figure 16:**
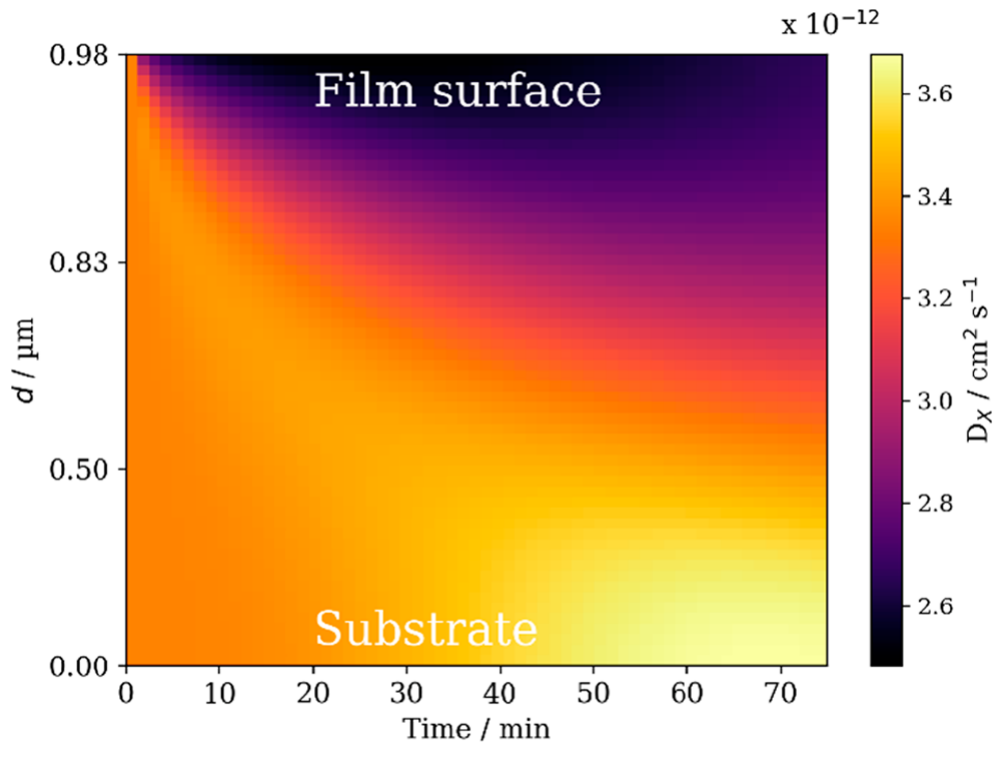
Evolution of ozone diffusivity throughout a 0.98 μm film during ozonolysis. [O_3_] = 77 ppm. *d*: distance from the film–substrate interface. Reproduced with permission from ref (4). Copyright 2022 the authors. Published by Copernicus Publications under a Creative Commons Attribution 4.0 International (CC BY 4.0) License.

The inclusion of a surface crust and making the diffusion coefficient of model components dependent on the bulk composition returned the best fits. The formation of the surface crust and its effect on molecular diffusion can be visualized by mapping the diffusion coefficient of ozone during a model run (*D*_*x*_ in Figure 16). A crust of dimer and trimer products inhibits the diffusion of ozone, limiting the reaction rate.

The optimized viscosity of the lamellar phase was ∼10^2^–10^3^ Pa s, putting it firmly in the semisolid regime.^18^ This value is also close to what we later determined for similar self-organized mixtures of oleic acid at ∼10^2^–10^4^ Pa s (Figure 9).^3^ We are therefore confident that our model captured the viscosity of the self-organized system well.

**Figure 17:**
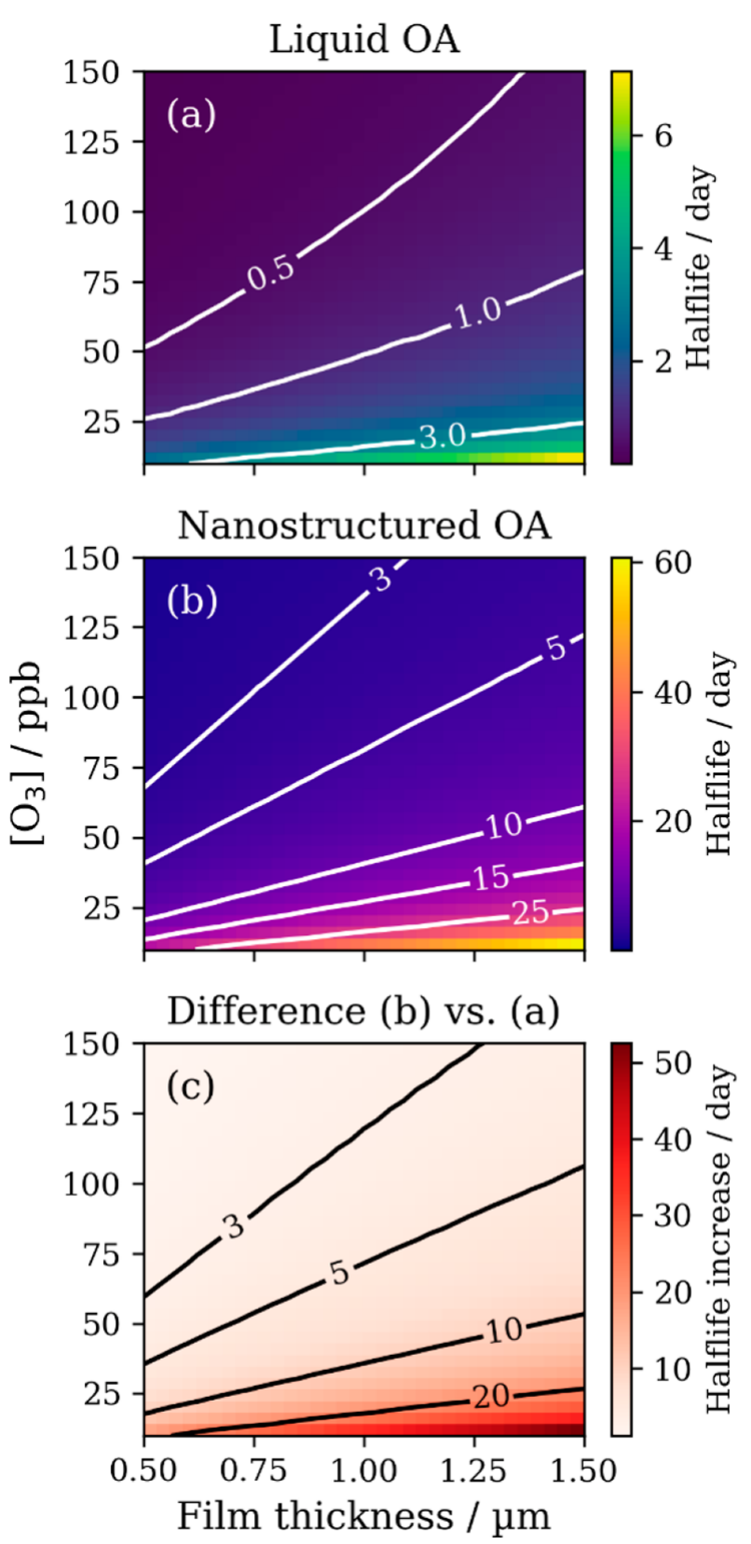
Plots of film half-life as a function of ozone concentration ([O_3_]) and film thickness. (a) Liquid oleic acid model. (b) Optimized lamellar-phase (nanostructured) oleic acid model. (c) Resulting increase in half-life due to nanostructure formation. Contours in each plot represent lines of constant half-life. Reproduced with permission from ref (4). Copyright 2022 the authors. Published by Copernicus Publications under a Creative Commons Attribution 4.0 International (CC BY 4.0) License.

By comparing the chemical half-lives of oleic acid in the liquid and nanostructured forms, we determined that for an ∼1 μm film at ∼30 ppb ozone there is an ∼10 day increase in the chemical half-life of oleic acid (Figure 17).

The impact of oleic acid nanostructure formation is therefore potentially significant. Oleic acid is unlikely to exist purely in a nanostructured form in the atmosphere. However, even if only a small portion of oleic acid forms such structures, the impact on the chemical lifetime could be significant and help explain the discrepancy between the longer atmospheric lifetime of oleic acid compared with laboratory predictions.^34,35^ This observation also extends to indoor surface coatings, which can contain fatty acids derived from cooking.^62^

## 7 Summary and Future Work

Most literature studies on oleic acid oxidation have focused on pure liquid oleic acid in its native form either as particles, deposited films, or monolayers,^29,63−65^ occasionally mixed with cosurfactants.^66^ Our work represents a new avenue, focusing on how the surfactant itself is organized and how this self-organization could impact the key aerosol processes of water uptake and chemical reaction outdoors and indoors.

Future interpretations of viscous indoor and outdoor aerosol phenomena should consider the possibility of surfactant self-organization, especially if the aerosol or film contains fatty acids such as those presented here.

Here, we list the most urgent future work in this field:

- *Experiments on more complex proxies and real atmospheric material*. Most of the work presented here has been on a simple oleic acid–sodium oleate proxy. This bottom-up approach has the advantage that we can determine the mechanisms through which our observations occur. The disadvantage is that these systems are not very realistic. Experiments on similar fatty acid surfactant systems (e.g., linoleic acid and stearic acid) are the next logical step, and the determination of the reactivity and hygroscopicity of specific phases is ongoing. Performing these experiments on atmospheric aerosol extracts would provide crucial insight into what happens in the atmosphere.
- *Experimental determination of physical parameters*. Our work has focused on chemical kinetics, which we can measure and model reasonably well. There remains a need to constrain models with direct measurements of viscosity and molecular diffusion in individual LLC phases. We have started to assess this (section 2). However, comprehensive rheological studies are still needed. Standard rheological^40^ and NMR techniques^67^ could address this. There is also a need to assess the optical properties of LLC phases, as they differ and can affect how reflective a cloud droplet could be, impacting the climate.^68^
- *Long-term aging experiments*. Experiments at large-scale facilities are limited to 2–4 days. This meant that our oxidation experiments involved ozone concentrations orders of magnitude greater than typical ambient values (ppm vs ppb levels) to measure kinetics in these lower-reactivity systems. Future work should look at longer-term aging and the inclusion of other atmospheric oxidants such as OH and NO_3_ radicals at atmospherically relevant concentrations.^66,69^ Oxidation by species such as chlorine, derived from cleaning products, would be of indoor air quality relevance.
- *Linking experiments and mechanistic models with larger-scale atmospheric models*. Kinetic multilayer modeling has helped us describe the impact of surfactant self-organization on processes at the aerosol and film levels. The recent development of MultilayerPy^58^ and methods to analyze the output of these models^70^ has increased the accessibility and interpretability of this kind of modeling. There is a need to efficiently link these computationally expensive models with large-scale atmospheric models that consider aerosols.^15^ Machine learning could be applied to this problem; for example the recent efforts of Berkemeier et al. have demonstrated the use of machine learning surrogate models based on KM-SUB outputs, reducing the computational cost.^57^

Our work has built a comprehensive platform, opening avenues for further study into the molecular arrangements of aerosol particles and film constituents. How these arrangements impact key atmospheric processes and affect the climate as well as indoor and outdoor air quality remains an open question that we have started to address for a small range of laboratory proxies. Our findings motivate future work on more complex proxy mixtures as well as atmospheric samples, linking to larger-scale modeling studies to expand our understanding of the abundance of self-organized structures in indoor and outdoor aerosols.
